# Statistical study of the causes of acacia tree deterioration in the Ha’il region

**DOI:** 10.7717/peerj.20067

**Published:** 2025-12-17

**Authors:** Alanazi Talal Abdulrahman, Khudhayr A. Rashedi, Tariq S. Alshammari

**Affiliations:** 1 Department of Mathematics, University of Ha’il, Hail, Saudi Arabia; 2 Scientific and Engineering Research Center, University of Ha’il, Hail, Saudi Arabia

**Keywords:** Acacia tree decline, Deforestation, Overgrazing, Climate change, Statistical analysis, Predictive modeling, Ha’il region, Conservation strategies

## Abstract

Acacia trees are vital to arid ecosystems, yet their decline in the Ha’il region of Saudi Arabia poses significant ecological and socio-economic concerns. This study employs a comprehensive statistical approach to identify the environmental and human-induced factors contributing to this deterioration. Data were collected from structured surveys, meteorological records, and satellite imagery. Analytical methods included descriptive statistics, correlation analysis, regression modeling, and chi-square automatic interaction detection (CHAID) classification. Results show that climate change—particularly increased temperatures and erratic rainfall—along with soil degradation, overgrazing, and urban expansion, are key drivers of acacia decline. Regression analysis revealed that drought severity (B = 3.721, *p* = 0.023) and urban growth (B = 3.365, *p* = 0.045) significantly predicted deterioration, explaining 17.5% of the variance (adjusted R^2^ = 0.175). The CHAID model identified urban expansion and overgrazing as critical risk factors, with deterioration scores ranging from 47.1 to 70.1 across subgroups. The study recommends strengthened conservation policies, improved irrigation, expanded protected areas, and increased public awareness to support acacia restoration efforts.

## Introduction

Acacia trees are critical components of arid and semi-arid ecosystems, particularly in regions like Ha’il, Saudi Arabia. These trees contribute significantly to biodiversity, offering habitats and food for various species while stabilizing soil and preventing erosion. Moreover, they serve local communities by providing fodder, wood, and medicinal resources (Zahran, 1999). The Ha’il region is home to two ecologically important varieties of *Vachellia gerrardii: var. negevensis* and *var. najdensis*. These varieties are highly adapted to arid environments and play a crucial role in sustaining biodiversity and maintaining ecological balance.

- *V. gerrardii var. negevensis* is typically found in gravelly wadi systems and dry, stony alluvial plains.
- In contrast, *V. gerrardii var. najdensis* is more commonly associated with sandy or loamy soils, often occupying open woodland areas and transitional desert zones.

Both types contribute to soil stability, serve as nitrogen fixers, and offer shade and forage for wildlife and livestock. Their presence reflects the adaptive capacity of native flora to the extreme environmental conditions of central Saudi Arabia, particularly in the Ha’il plateau (Llewellyn et al., 2011).

In the Ha’il region, *V. gerrardii* is found in two ecologically significant varieties: var. negevensis and var. najdensis. The negevensis variety typically inhabits arid, gravelly wadis and stony plains, whereas najdensis is more commonly associated with sandy or loamy soils in open woodlands and desert transition zones. These varieties play an essential role in sustaining local ecosystems by providing forage, shade, and shelter for wildlife and livestock. Due to their sensitivity to environmental pressures, they are also considered important indicators of ecological change (Llewellyn et al., 2011).

The selection of key variables in this study, namely drought severity, urban development, overgrazing, and soil degradation, was guided by existing literature that highlights their role in tree decline across arid landscapes (Chesson & Huntly, 1997; Alanazi et al., 2022; Zhang et al., 2003). Drought reduces water availability, weakening trees and increasing their vulnerability to pests and disease. Urbanization contributes to habitat loss and fragmentation, while overgrazing hinders seedling regeneration. Additionally, salinity and erosion degrade soil quality, further impeding acacia growth. These factors collectively offer a holistic view of the environmental and human-driven pressures affecting Acacia populations in the Ha’il region.

However, in recent years, the health and population of acacia trees in the Ha’il region have been declining, which threatens ecological stability and local livelihoods.

The environmental challenges in the Ha’il region, such as high temperatures, limited rainfall, and poor soil conditions, exacerbate the vulnerability of these trees (El-Rawy et al., 2023). In addition to these natural stressors, human activities like urban expansion, overgrazing, and deforestation have further intensified the degradation of acacia populations. This situation necessitates a detailed statistical analysis to identify the primary factors contributing to their decline and to propose conservation strategies that are both effective and evidence- based (Chesson & Huntly, 1997). Acacia trees are fundamental to the ecosystems of arid and semi-arid regions, where they contribute to biodiversity, prevent soil erosion, and support local communities. These trees have adapted to harsh environmental conditions, making them essential for sustaining life in water-scarce areas. Additionally, acacia trees enrich the soil by fixing nitrogen through symbiotic relationships with rhizobia bacteria, a process that is particularly valuable in nutrient-deficient soils (Zahran, 1999).

Various environmental factors, such as inconsistent rainfall, rising temperatures, and poor soil quality, significantly affect the growth and survival of acacia trees. Research has shown that prolonged droughts and erratic rainfall patterns are leading contributors to vegetation loss in arid regions (Chesson & Huntly, 1997). Furthermore, the increasing frequency of heatwaves and higher average temperatures due to climate change worsens water stress, making acacia trees more vulnerable to diseases and pests. Soil salinity is another critical challenge for acacia trees in arid areas. High salinity levels prevent trees from effectively absorbing water, which negatively impacts their growth and productivity. Studies leveraging remote sensing and Geographic Information System (GIS) technology have identified soil salinity and erosion as major issues in arid environments, including the Ha’il region (El-Rawy et al., 2023).

Human activities, such as urbanization, overgrazing, and deforestation, are key factors driving the deterioration of acacia populations. Urban development fragments habitats, while overgrazing damages seedlings and prevents natural regeneration (Chesson & Huntly, 1997). Additionally, the overharvesting of acacia trees for fuelwood and agricultural expansion reduces tree populations and disrupts the ecosystem.

Acacia trees in the Negev Desert serve as keystone species, increasing plant diversity and soil nutrient levels beneath their canopies, though the impacts vary depending on tree health and water stress. While trees improve soil quality, particularly in larger and healthier individuals, greater water stress and mortality are linked to changes in species composition and possibly higher soil salinity (Munzbergova & Ward, 2002). *Acacia nilotica*-based Silvi pastoral systems in India’s damaged Lower Himalayan region reduced soil, water, and nutrient losses while enhancing soil microbial characteristics; however, intercropped grasses had different degrees of negative impact on Acacia development. Acacia + *Vetiveria zizanioides* outperformed the other systems studied in terms of resource conservation and economic return, producing the greatest biomass and having the best financial viability (Yadav et al., 2014). A study of 1,540 *Acacia xanthophloea* trees in Ol Pejeta Conservancy indicated that enclosed regions had much higher tree densities than open areas, with elephants being responsible for the bulk of tree damage (54.55%). Herbivory had no substantial impact on seedling regeneration, but it did disturb population structure, emphasizing the need for expanded closures and improved management of browser densities (Muhati & Abdillahi, 2016). The use of radiocarbon dating and growth ring analysis on *Acacia erioloba* in the Kgalagadi Transfrontier Park demonstrated a significant relationship between age and stem circumference, which facilitated assessments of age structure. Populations located in the Nossob Riverbed exhibited low recruitment rates, whereas those in the dune fields displayed a more robust age distribution, indicating varying regeneration dynamics that are not influenced by flooding (Muhati & Abdillahi, 2016). Research on *Acacia mearnsii* timber revealed that tannin extract greatly improved resistance against white rot fungus, demonstrating preservative properties like those of the widely used but harmful copper chromium boron (CCB) mixture. These results indicate that tannin could serve as a viable natural option for improving wood durability while minimizing environmental and health hazards (Steenkamp et al., 2008). A research study conducted on *Acacia tortilis* subsp. Raddiana in the arid regions of Tunisia indicated that the growth of these trees enhances soil quality by increasing microbial biomass, organic carbon content, and enzyme activity, while also lowering the carbon: nitrogen (C: N) ratio and metabolic stress. Nevertheless, heavy grazing adversely impacted these benefits, emphasizing the species’ capacity for soil restoration and the significance of managing grazing practices (Silveira et al., 2017). Research on *Acacia mangium* stands indicated that the intensity of thinning had a notable impact on litter dynamics, with untinned areas generating the highest amounts of litter and nutrient contributions, while plots that were heavily thinned exhibited quicker litter decay because of changes in the microenvironment. These findings emphasize a trade-off between the amount of litter produced and the rate of decomposition, where untinned stands promote carbon storage and nutrient conservation (Fterich, Mahdhi & Mars, 2012).

Recent studies have increasingly documented the susceptibility of Acacia species to both environmental and human-induced stressors in arid and semi-arid ecosystems. In the Negev Desert, for instance, *Acacia raddiana* serves as a keystone species, and its decline has been associated with decreased biodiversity and soil nutrient depletion, particularly under prolonged drought conditions (Munzbergova & Ward, 2002). In Tunisia, *Acacia tortilis* subsp. Raddiana has been shown to enhance soil microbial biomass and organic carbon levels, but these positive effects are significantly diminished in heavily grazed areas, reflecting its sensitivity to anthropogenic pressures (Field, 2018). In India, silvipastoral systems incorporating *Acacia nilotica* have improved land productivity and curbed erosion in degraded zones; however, their success is closely linked to effective grazing control and sustainable land-use practices (Yadav et al., 2014).

Complementing these regional findings, more recent global research (Alemayehu, Suarez-Minguez & Rosette, 2024; Liiv et al., 2025) underscores how climate change, along with pressures like urban expansion, overgrazing, and land conversion, has accelerated tree mortality and ecological imbalance in dryland environments. These insights reinforce the rationale behind selecting key variables such as drought severity, grazing intensity, soil salinity, and urbanization in our analysis of acacia decline in the Ha’il region, while situating this case within a wider global ecological context.

In Algeria and broader Saharan contexts, recent ecological assessments (Bouzekri et al., 2023) have documented the negative effects of overexploitation and salinization on Acacia regeneration, highlighting the role of anthropogenic stressors in landscape degradation. Moreover, a comparative study from the Sinai Peninsula emphasized the ecological sensitivity of desert Acacias to both natural and human-induced stressors, underscoring the necessity for region-specific management interventions (Moustafa et al., 2015). These findings collectively support the need for integrated conservation strategies tailored to arid ecosystems experiencing parallel threats.

Although the importance of acacia trees is well known, there is a lack of local studies that measure how and why these trees are declining. Existing research often overlooks the combination of ecological and social factors, which are both essential to understanding the problem fully. This gap in knowledge makes it hard to create targeted, effective conservation strategies for the Ha’il region.

This study aims to:

1. Measure the extent of acacia tree decline using statistical tools.
2. Study how climatic factors (such as rainfall and temperature) relate to tree health.
3. Evaluate the impact of human activities such as grazing and land development.
4. Develop models to predict future risks and guide conservation actions.

Hypothesis: Environmental conditions and human behavior significantly influence the decline of acacia trees in Hail.

This research is urgent because the ongoing loss of acacia trees threatens the region’s environment and economy. The study provides new insights by combining field data, public opinion surveys, and advanced statistical methods. Its findings will help decision-makers and local communities take effective steps to protect these important trees and manage land more sustainably.

This article is structured into several sections. Following the introduction, the literature review discusses prior studies on acacia ecology and the factors contributing to their decline. The methodology section outlines the data collection process, variables studied, and statistical techniques employed. The results and discussion section presents key findings and interprets their significance. Finally, the conclusion summarizes the research outcomes and offers recommendations for conservation strategies.

## Methodology

### Study area

This research was conducted in the Ha’il region of Saudi Arabia, an arid zone characterized by low rainfall, high temperatures, and limited water availability. The region is home to acacia trees that play a critical ecological and socio-economic role. The study area was selected due to the environmental challenges and the observed decline in acacia populations, making it a suitable location for investigating the factors contributing to their deterioration.

**Figure 1:**
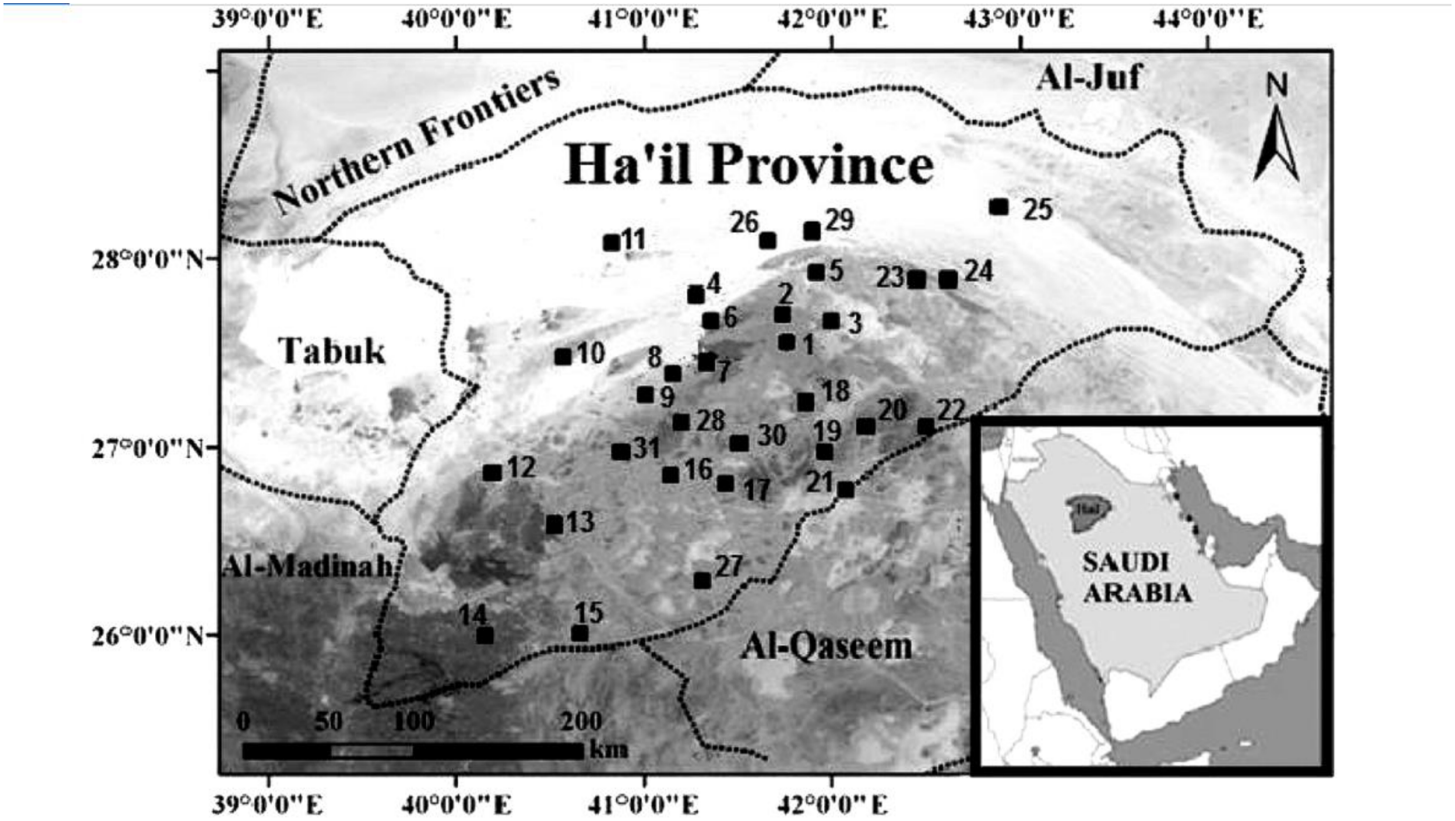
The 31 locations within Ha’il Province where data collection was conducted. These locations were selected based on the ecological presence of acacia trees and environmental stress indicators, providing a spatial overview of the study area.

According to Alshammari, Busais & Ibrahim (2017), the Ha’il Region is situated in the northern central part of Saudi Arabia. The area of the region is 118,232 square kilometers. Ha’il is situated in the center of the region. It is the only significant city in the area. Located 600 km from Riyadh, 450 km from Madinah, and 650 km from Tabuk, it is easily accessible from other regional centers. Ha’il has a temperate climate due to its elevation of 915 m above sea level. The study was conducted across 31 locations in Ha’il Province (Fig. 1) based on the mapping provided by Alshammari, Busais & Ibrahim (2017).

According to Climate Data (2025), over the past 20 to 50 years, Ha’il—like much of the Arabian Peninsula—has experienced significant climate changes, particularly in rising temperatures. Although detailed long-term records for Ha’il are limited, broader regional data indicate a consistent warming trend consistent with global climate change. For example, the average national temperature in Saudi Arabia from 1961 to 1990 was approximately 24.7 °C, and temperatures have steadily increased in the decades since. Today, Ha’il records an average temperature of approximately 22.7 °C (72.8 °F).

Rainfall levels in Ha’il have remained low and stable over the years, in keeping with its desert climate. The region receives an average of only 81 mm (3.2 inches) of rain annually, a figure that has seen little change over the decades.

Summer, which runs from June to September, is extremely hot, with temperatures often exceeding 40 °C (104 °F). In contrast, winters are characterized by mild temperatures, with nighttime temperatures sometimes dropping below 10 °C (50 °F). For those planning a visit, April, May, and October offer more comfortable conditions and are considered the best times to explore the region.

Furthermore, we improved the study area’s environmental profile by providing a more thorough analysis of the soil’s physical and chemical properties, which are crucial for understanding Acacia species’ ecological dynamics. The soils in the Ha’il region are mostly sandy to loamy, with minimal organic matter, limited moisture retention, and high alkalinity (pH > 7.5). Alkaline environments, high calcium carbonate levels, and moderate cation exchange capacity (CEC) have a substantial impact on nutrient availability and root development in Acacia trees. Including these soil factors gives a more rigorous ecological context for examining acacia degradation patterns in the area. Including these soil parameters provides a more robust ecological context for analyzing acacia degradation patterns in the region (Farrag, 2012; Salman, 2015).

These climate trends highlight the growing importance of environmental awareness and adaptation strategies in arid regions like Ha’il, especially considering ongoing global climate change.

### Data collection

#### Opinion-based questionnaires

Data for the study were collected using structured opinion-based questionnaires distributed to residents, landowners, and environmental specialists in the Ha’il region. These questionnaires aimed to capture participants’ perceptions and knowledge about:

- Environmental factors: the extent of the impact of drought and lack of rain, impact of climate change (such as rising temperatures), impact of desertification, Impact of sandstorms, effect of soil erosion, Impact of flash floods, the effect of high salinity in soil and water and impact of low quality of water used for irrigation.
- Human activities: the effect of overcutting, Effect of overgrazing, impact of urban expansion, the impact of agricultural expansion, impact of industrial activities (such as factories and major projects), impact of mining and exploitation of natural resources, the impact of unregulated ecotourism, the impact of the use of heavy equipment on the surrounding areas, impact of air pollution due to human activities and overhunting birds that feed on insects and worms.

Overall, the data consists of 17 key factors (VAR1 to VAR17), each representing the causes of Acacia tree deterioration. Responses are recorded on the Likert scale (1 to 5), where:

➢ 1 = No effect
➢ 2 = Weak effect
➢ 3 = Medium effect
➢ 4 = Great effect
➢ 5 = Very big effect

• What is the percentage of decline of acacia trees in the Hail region based on the factors mentioned above?
• What solutions do you think are appropriate to reduce the deterioration of acacia trees?
  - Improving irrigation techniques.
  - Imposing strict laws on illegal cutting.
  - Organizing grazing.
  - Expansion of protected areas.
  - Planting more acacia trees.
  - Educating the community about the importance of acacia trees.
  - Confronting desertification through government programs.

To investigate local perceptions regarding the decline of Acacia trees, a structured questionnaire was designed and reviewed by experts in ecology and statistics to ensure validity and clarity. The survey included both closed-ended items—using a five-point Likert scale (ranging from 1 = no effect to 5 = very strong effect)—and open-ended questions, allowing respondents to elaborate on their views. The 17 closed-ended items (VAR1–VAR17) captured various environmental and anthropogenic factors suspected to influence tree deterioration.

A purposive sampling approach was used to target individuals with first-hand knowledge of the region’s Acacia ecosystems. Participants were selected from 31 locations across the Ha’il region and included farmers, pastoralists, landowners, and environmental practitioners with at least five years of residency in the area. This method ensured responses were grounded in direct experience and ecological familiarity.

Of the 300 questionnaires distributed, 234 were returned fully completed, resulting in a 78% response rate indicative of strong community engagement and interest in environmental issues affecting the region. Table 1 summarizes the questionnaires, which incorporated both closed-ended and open-ended questions to gather comprehensive feedback and allow respondents to express their unique perspectives.

**Table 1:** The response questionnaires.

Variable	Questionnaires	Response
**E**	What is the percentage of decline of acacia trees in the Hail region based on the factors mentioned above	Percentage number
**VAR1**	The extent of the impact of drought and lack of rain	-Very big effect -Great effect -Medium effect -Weak effect -No effect
**VAR2**	Impact of climate change (such as rising temperatures)	
**VAR3**	Impact of desertification	
**VAR4**	Impact of sandstorms,	
**VAR5**	Effect of soil erosion,	
**VAR6**	Impact of flash floods,	
**VAR7**	The effect of high salinity in soil and water	
**VAR8**	Impact of low quality of water used for irrigation	
**VAR9**	The effect of overcutting	
**VAR10**	Effect of overgrazing,	
**VAR11**	Impact of urban expansion,	
**VAR12**	The impact of agricultural expansion,	
**VAR13**	Impact of industrial activities (such as factories and major projects),	
**VAR14**	Impact of mining and exploitation of natural resources,	
**VAR15**	The impact of unregulated ecotourism,	
**VAR16**	The impact of the use of heavy equipment on the surrounding areas,	
**VAR17**	Impact of air pollution due to human activities and overhunting birds that feed on insects and worms	

In this survey, which examined public awareness and attitudes toward acacia conservation, the sample consisted of residents from rural communities in the Hail region of Saudi Arabia, where acacia species are native. Participants ranged in age from 18 to 70 and included farmers, herders, teachers, and local officials.

Inclusion criteria required participants to have lived in the area for at least five years and to have a basic knowledge of the local flora. Exclusion criteria included individuals under the age of 18 and those who had recently moved to the area, ensuring that the survey was limited to individuals with long-term exposure to the local environment.

A purposive sampling strategy was used, selecting individuals from communities close to key acacia habitats. This approach was chosen to ensure that the sample included people with relevant experience and knowledge of the trees. The rationale for this method was to gather detailed insights from those directly affected by and familiar with acacia ecosystems, rather than from the general population.

#### Supplementary environmental and anthropogenic data

To enhance the reliability of findings, the study triangulated perception data with quantitative environmental datasets:

• Meteorological Records:
Climate data—such as annual rainfall, temperature averages, and seasonal variability were obtained from the Saudi General Authority for Meteorology and Environmental Protection and cross-validated with historical climate trends from publicly available climate databases (Climate Data, 2025; General Authority for Meteorology and Environmental Protection, 2021).
• Satellite Imagery:
Land cover and urban expansion patterns were analyzed using Landsat imagery (NASA/USGS, 2023), MODIS vegetation indices (NASA, 2023), and elevation data from SRTM (CGIAR Consortium for Spatial Information (CGIAR-CSI), 2008). These datasets were processed using GIS tools to generate maps of grazing intensity, land degradation, and deforestation trends in the Ha’il region.

These spatial data were used to cross-reference community responses and to identify visible patterns of Acacia decline, allowing for a more comprehensive understanding of ecological stressors.

To understand the environmental and human-induced factors contributing to the decline of acacia trees in the Ha’il region, this study relied on a combination of data sources, including questionnaires and insights from existing research.

### Environmental factors

Data regarding rainfall, temperature, and soil conditions were collected through meteorological records and supplemented by information gathered *via* questionnaires distributed to residents, landowners, and environmental experts. Participants were asked to share their perceptions of how these environmental variables impact the health of acacia trees in the region.

Findings from Alanazi et al. (2022) provided additional context for interpreting environmental impacts. Their study identified acacia trees in dry environments, such as plateaus, that face heightened vulnerability to drought and pest infestations. Prolonged droughts weaken trees, reducing their ability to fend off pests like the xylophagous beetle Steraspis speciosa. This beetle’s larvae burrow into the bark, causing severe damage to the xylem and phloem, which disrupts water and nutrient transport and leads to tree mortality. The insights from this research supported questionnaire responses indicating that limited water availability exacerbates tree decline in the Ha’il region.

### Anthropogenic factors

The role of human activities, including grazing, urbanization, and firewood collection, was examined through both satellite imagery and questionnaire responses. Respondents highlighted overgrazing as a significant issue, particularly in unprotected areas where young acacia trees are unable to regenerate. Additionally, urban expansion and firewood harvesting were reported as key contributors to habitat degradation.

The observations align with the findings of Alanazi et al. (2022), who noted that areas exposed to anthropogenic pressures exhibit higher rates of acacia decline. The combination of human activities and environmental stressors creates a compounding effect, further weakening tree health and increasing vulnerability to pest infestations.

### Pest dynamics and their interaction with other factors

Responses from questionnaires indicated a growing awareness of pest activity as a factor in tree decline. Participants noted the presence of beetle infestations in certain areas, corroborating the findings of Alanazi et al. (2022), who documented that Steraspis speciosa preferentially targets older trees in drier habitats. The study also highlighted that pest infestations are more severe in regions with limited soil moisture, suggesting an interplay between environmental stress and pest dynamics.

By integrating questionnaire data with insights from the literature, this study provides a comprehensive understanding of the environmental and anthropogenic factors driving acacia tree decline in the Ha’il region.

### Data analysis

#### Descriptive statistics

Descriptive statistical methods were used to summarize and analyze the questionnaire responses. Frequencies, percentages, and averages were calculated to provide an overview of the perceptions related to tree health, environmental factors, and human activities.

Descriptive statistics are widely used in ecological studies to summarize and simplify complex datasets, offering a clear overview of trends and patterns in environmental and anthropogenic data. Commonly employed measures include the mean, standard deviation, and frequency. The sample mean provides a measure of central tendency, calculated as:

$\; \bar x = \; \displaystyle{{\mathop \sum \nolimits_{i = 1}^n {x_i}} \over n}$

where:

$\bar x$ = sample mean,

$\mathop \sum \nolimits_{i = 1}^n {x_i}$ = individual data points,

*n* total number of data points.

The sample standard deviation assesses the variability of data, expressed as:

$s = \sqrt {\displaystyle{{\mathop \sum \nolimits_{i = 1}^n {{\left( {{x_i} - \bar x} \right)}^2}} \over {n - 1}}}.$

Hasel, Bügelmayer-Blaschek & Formayer (2023) emphasized that descriptive statistics are crucial for summarizing climatic and anthropogenic factors, providing the groundwork for advanced analyses such as correlation or regression. For example, summarizing rainfall data across seasons highlights variability, while frequency distributions of questionnaire responses capture human perceptions of ecosystem health.

#### Correlation analysis

Correlation analysis was performed to evaluate the relationships between participants’ perceptions of environmental factors (*e.g*., rainfall, temperature) and their observations of tree health. This analysis helped identify the key drivers of Acacia deterioration based on respondents’ feedback.

Correlation analysis is a statistical tool used to examine the strength and direction of relationships between two variables, often quantified using Pearson’s correlation coefficient (r). The equation for r is:

$r = \; \displaystyle{{\mathop \sum \nolimits_{i = 1}^n \left( {{X_i} - \bar X} \right)\left( {{Y_i} - \bar Y} \right)} \over {\sqrt {\left[ {\mathop \sum \nolimits_{i = 1}^n {{\left( {{X_i} - \bar X} \right)}^2}} \right]\left[ {\mathop \sum \nolimits_{i = 1}^n {{\left( {{Y_i} - \bar Y} \right)}^2}} \right]} }}$

where:

-
${X_i},\; {Y_i}$ individual data points for variables X and Y,
-
$\bar X,\; \bar Y$ means of X and Y, respectively.
- Range of r:
- r > 0: Positive correlation (*e.g*., rainfall and tree health).
- r < 0: Negative correlation (*e.g*., temperature and tree health).
- r = 0: No correlation.

Amlani et al. (2023) used correlation analysis to examine the relationship between temperature fluctuations and forest mortality rates, identifying significant negative correlations. This method is particularly effective for exploring environmental and anthropogenic factors, such as how grazing intensity correlates with reduced tree regeneration.

#### Regression analysis

Multiple regression models were developed to assess the combined effects of environmental and anthropogenic factors on acacia populations. These models helped quantify the influence of different variables and provided a clearer understanding of their impact on tree health.

Regression analysis is essential for modeling the influence of one or more independent variables on a dependent variable.

• Simple linear regression: Examines the relationship between a single predictor (X) and a response (Y):
$y = {a_0} + {a_1}x + \; \rm \epsilon$
where:
$y$ = dependent variable (*e.g*., tree health),
X = independent variable,
${a_0}$ = interception,
${a_1}$ = slope of the regression line,
$\rm \epsilon$ = error term.
This model quantifies the combined effects of environmental (*e.g*., rainfall, temperature) and anthropogenic factors (*e.g*., grazing intensity). The distinction between the two:
• Simple linear regression: Involves one independent variable predicting a dependent variable.
• Multiple linear regression: Involves two or more independent variables predicting a dependent variable.

According to Hasel, Bügelmayer-Blaschek & Formayer (2023), regression analysis is particularly effective for understanding complex ecological systems where multiple variables interact. For example, regression can reveal how drought exacerbates pest activity, further weakening tree health.

#### Predictive modeling

The data obtained from the questionnaires and supplementary sources was used to build predictive models. These models aimed to estimate future risks to acacia populations and identify areas requiring immediate conservation efforts.

Predictive modeling uses historical data and statistical algorithms to forecast future risks and outcomes, making it a vital tool for conservation efforts.

- Decision trees: A tree-based algorithm that splits data into subsets based on decision rules:

Decision rule: If ${X_i} \le t$, assign to region 1; otherwise, region 2.

Where:

${X_i}$ predictor variable,

t threshold value.

The chi-squared automatic interaction detector (CHAID) model was employed to identify the most influential predictors of Acacia decline among the 17 variables. CHAID is a non-parametric, tree-based classification technique that segments data into mutually exclusive subgroups based on statistically significant splits. It is particularly useful in exploratory ecological research involving categorical or ordinal predictors.

Rationale for Use:

- CHAID accommodates multi-level categorical variables without requiring distributional assumptions, making it ideal for Likert-scale survey data.
- It allows for the interpretation of interaction effects, enabling researchers to identify specific combinations of environmental and anthropogenic stressors that increase risk.
- The method provides actionable thresholds, helping identify critical conditions (*e.g*., high urban expansion + poor water quality) that define high-risk zones.

In this study, the CHAID model identified urban expansion (VAR13) and overgrazing (VAR1) as the two most decisive variables, which segmented the dataset into interpretable subgroups with differing mean deterioration scores (from 47.1 to 70.1). This output supports targeted conservation strategies by highlighting priority intervention areas.

Alemayehu, Suarez-Minguez & Rosette (2024) demonstrated the effectiveness of random forest models in predicting species distributions under climate change scenarios. These models are particularly valuable for identifying high-risk areas for acacia decline, integrating climatic and human activity variables to guide conservation strategies.

However, the study lacks detailed procedures for model calibration, such as parameter tuning or alternative estimation techniques beyond default statistical software settings. Sensitivity analysis was not conducted to evaluate how variations in input variables influence output predictions. Additionally, no formal validation procedures, such as split sample testing or cross-validation were applied to assess the model’s generalizability. The absence of uncertainty analysis, including confidence intervals for model estimates and error propagation, limits the robustness and transparency of the predictive models. These limitations may reduce the reproducibility of the findings and highlight the need for more rigorous methodological depth in future research.

### Ethical considerations

The study followed ethical guidelines to ensure the integrity of the research process. After obtaining approval from the Research Ethics Committee, the questionnaire was distributed to the target group. Research Ethics Committee (RCE) at the University of Ha’il reviewed and approved this study on 03/02/2025, with research number H-2025-587. Verbal and written consent were obtained from the participants before data collection.

### Limitations

The study faced some limitations, including reliance on self-reported data, which may be subject to personal biases or inaccuracies. Additionally, the absence of direct field measurements restricted the ability to validate participants’ perceptions. Future research could integrate opinion-based data with physical assessments for a more robust analysis.

## Results and discussion

### The results of the general data analysis

**Figure 2:**
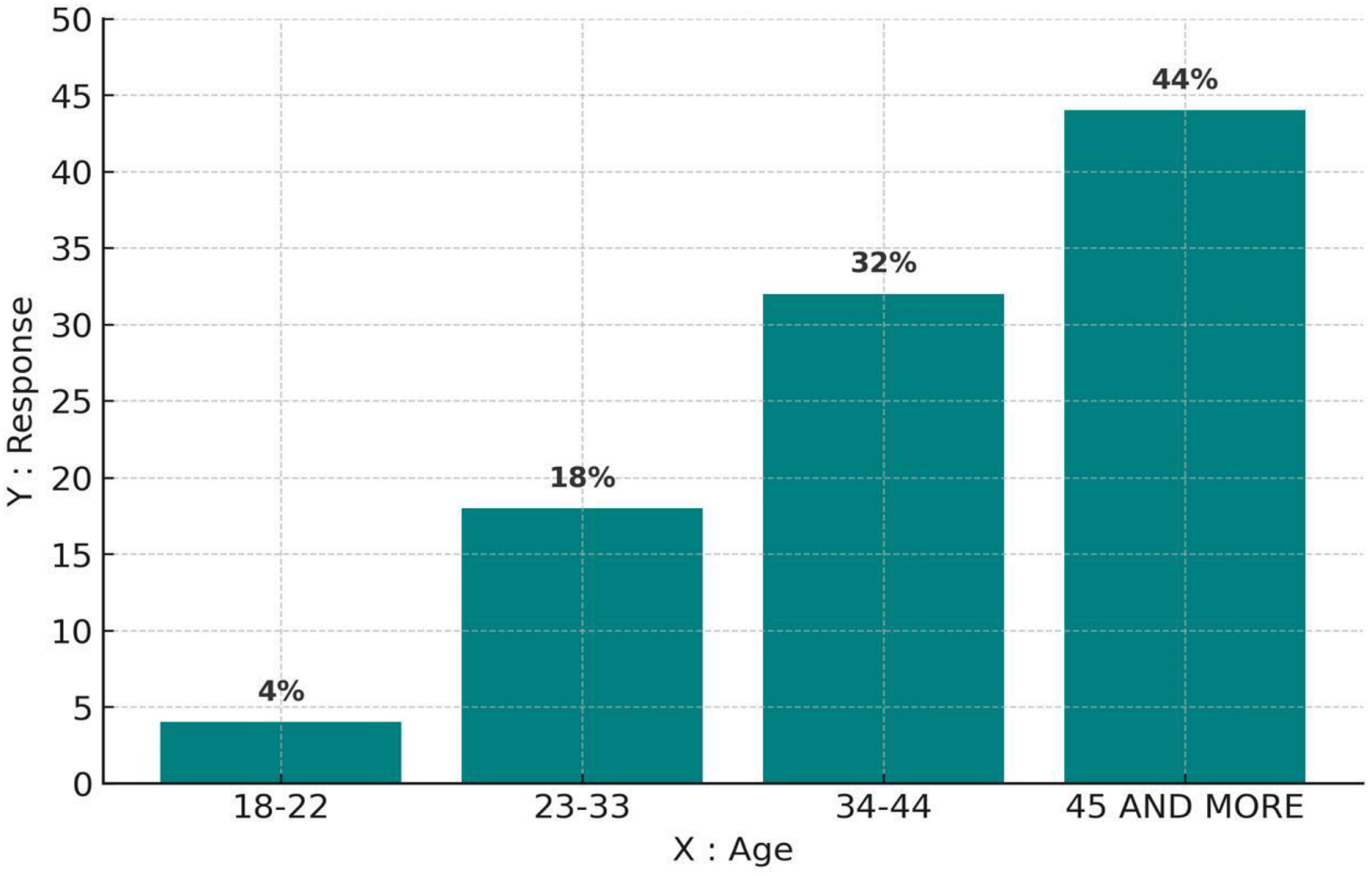
The distribution of respondents by age group. The highest participation was among individuals aged 45 and above, indicating a strong engagement from older residents who may have greater familiarity with environmental changes in the region.

Figure 2 presents the distribution of responses based on age categories. The highest engagement was recorded among individuals aged 45 and above (44%), followed by the 34–44 age group (32%). In contrast, respondents aged 23–33 accounted for 18%, while the youngest group, 18–22 years old, contributed only 4%. These results suggest that older participants were more inclined to complete the questionnaire, possibly due to greater interest or availability, whereas younger respondents may require more targeted outreach.

**Figure 3:**
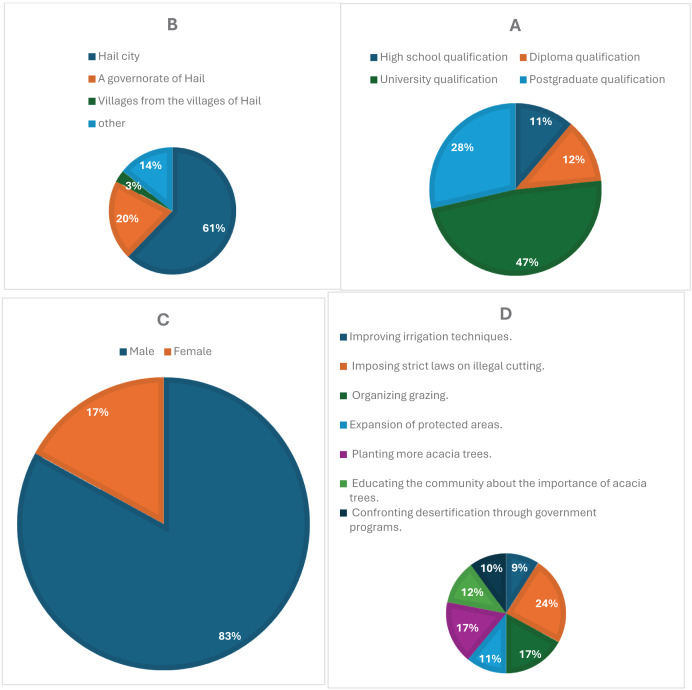
(A) Educational levels of participants, with university and postgraduate respondents showing the highest engagement. (B) Residential distribution, highlighting a concentration of responses from urban areas, particularly Hail city. (C) Gender distribution, showing a predominance of male respondents (83%). (D) Public suggestions for conservation, including support for anti-cutting laws, organized grazing, and tree planting initiatives.

Figure 3A illustrates how survey participation varied across different educational backgrounds. Respondents with a university degree made up the largest proportion (47%), followed by those with a postgraduate qualification (28%). In comparison, diploma holders accounted for 12%, while individuals with only a high school qualification represented 11%. The higher response rates among university-educated participants suggest a potential correlation between academic background and engagement in the study.

Figure 3B compares survey participation based on respondents’ places of residence. Most responses came from Hail city (61%), while governorates in Hail contributed 20%. Participation from villages within Hail was considerably lower (3%), whereas 14% of respondents reported living in other locations. The data suggests that urban dwellers were more likely to engage with the survey, whereas those in rural areas may have encountered accessibility challenges or lower awareness of the study.

In Fig. 3C, gender-based participation in the survey shows a clear disparity, with male respondents making up 83% of the sample, while female participation was significantly lower at 17%. This imbalance could be attributed to differences in interest, accessibility, or social factors influencing engagement. To ensure a more representative gender distribution in future studies, additional efforts may be needed to encourage female participation.

Figure 3D presents the most frequently suggested strategies for protecting acacia trees. The most widely supported measure was enforcing strict regulations against illegal tree cutting (24%), followed by both organized grazing (17%) and increasing tree-planting efforts (17%). Additionally, expanding protected areas (11%) and enhancing irrigation methods (9%) were also proposed. These findings reflect a strong awareness of environmental conservation and highlight key areas for policy development and ecological management.

### The results of the regression analysis

Table 2 summarizes the descriptive statistics for the variables included in the study. The dependent variable, VAR18, has a mean of 53.3205 with a standard deviation of 23.25940, and the coefficient of variation (CV) is 43.62187151% across 234 observations. Independent variables, such as VAR1 through VAR17, show varying means and standard deviations, reflecting their unique distributions and roles within the model.

**Table 2:** The descriptive statistics for the variables.

	Mean	Std. deviation	The coefficient of variation (CV) %	N
**E**	53.3205	23.25940	43.62187151	234
**VAR1**	3.7393	1.14431	30.60225176	234
**VAR2**	3.3761	1.17358	34.7614111	234
**VAR3**	3.8034	1.07439	28.2481464	234
**VAR4**	3.3034	1.24896	37.8083187	234
**VAR5**	3.5085	1.18735	33.84209776	234
**VAR6**	3.1538	1.36237	43.19772972	234
**VAR7**	2.9615	1.20203	40.5885531	234
**VAR8**	2.9573	1.25259	42.35586515	234
**VAR9**	4.6496	0.66573	14.31800585	234
**VAR10**	4.2521	0.98077	23.06554408	234
**VAR11**	4.0342	1.13040	28.02042536	234
**VAR12**	3.6239	1.27859	35.28215458	234
**VAR13**	3.7607	1.25435	33.35416279	234
**VAR14**	3.7094	1.20129	32.38502184	234
**VAR15**	3.4188	1.29535	37.88902539	234
**VAR16**	3.7521	1.18580	31.6036353	234
**VAR17**	3.5385	1.18675	33.53822241	234

Table 3 presents the Pearson correlation coefficients among the variables. VAR18 demonstrates moderate positive correlations with several independent variables, including VAR1 (r = 0.287, *p* < 0.00) and VAR4 (r = 0.325, *p* < 0.00), indicating potential predictive relationships.

**Table 3:** The Pearson correlation coefficients among the variables.

Pearson correlation	E		Sig. (1-tailed)
**E**	1.000	**E**	
**VAR1**	0.287	**VAR1**	0.000
**VAR2**	0.260	**VAR2**	0.000
**VAR3**	0.231	**VAR3**	0.000
**VAR4**	0.325	**VAR4**	0.000
**VAR5**	0.284	**VAR5**	0.000
**VAR6**	0.203	**VAR6**	0.001
**VAR7**	0.188	**VAR7**	0.002
**VAR8**	0.136	**VAR8**	0.019
**VAR9**	0.115	**VAR9**	0.040
**VAR10**	0.105	**VAR10**	0.054
**VAR11**	0.148	**VAR11**	0.012
**VAR12**	0.159	**VAR12**	0.007
**VAR13**	0.213	**VAR13**	0.001
**VAR14**	0.160	**VAR14**	0.007
**VAR15**	0.192	**VAR15**	0.002
**VAR16**	0.188	**VAR16**	0.002
**VAR17**	0.242	**VAR17**	0.000

Table 4 provides a regression model summary, regression coefficient, and Pearson correlation. The adjusted R^2^ value is 0.175, indicating that approximately 17.5% of the variance in the dependent variable is explained by the independent variables. The model’s overall F-statistic (F = 2.695, *p* < 0.000) demonstrates statistical significance. To evaluate potential multicollinearity among predictor variables, a correlation matrix was created that included all independent and dependent variables. The regression model predicts a dependent variable based on 17 independent variables (VAR1 to VAR17) with a constant term.

$Predicted\; Value = 9.54 + {\rm \; }3.72{\rm \; VAR}1 + \ldots + 2.20{\rm \; VAR}17 + \rm \epsilon$

**Table 4:** The regression model summary.

Model	R	R square	Adjusted R square	Std. error of the estimate	Change statistics				
					R square change	F change	df1	df2	Sig. F change
1	0.418	0.175	0.110	21.94182	0.175	2.695	17	216	0.000

Model	Regression coefficient
**(Constant)**	9.546
**VAR1**	3.721
**VAR2**	0.632
**VAR3**	−0.254
**VAR4**	3.365
**VAR5**	1.703
**VAR6**	0.836
**VAR7**	0.809
**VAR8**	−2.800
**VAR9**	2.324
**VAR10**	−0.723
**VAR11**	−0.094
**VAR12**	−0.199
**VAR13**	0.877
**VAR14**	−2.222
**VAR15**	0.685
**VAR16**	0.805
**VAR17**	2.203
**Group**	**Correlation**
**VAR5 and VAR6**	0.522
**VAR7 and VAR8**	0.666
**VAR11 and VAR12**	0.674
**VAR12 and VAR13**	0.575
**VAR13 and VAR14**	0.777
**VAR14 and VAR15**	0.509
**VAR15 and VAR16**	0.540
**VAR16 and VAR17**	0.595

Variables with large positive coefficients suggest a strong positive relationship with the dependent variable:

- VAR1: 3.72
- VAR4: 3.36
- VAR9: 2.32
- VAR17: 2.20
- VAR5: 1.70
- VAR13: 0.87
- VAR6, VAR7, VAR16, VAR15: Moderate positive values (around 0.6 to 0.8)

Variables with negative coefficients suggest an inverse relationship:

- VAR8: −2.80(strong negative influence)
- VAR14: −2.22
- VAR10: −0.72
- VAR3, VAR12, VAR11: Slightly negative impact.

Multicollinearity diagnostics based on Pearson correlation coefficients revealed notable intercorrelations among several predictors. Specifically, strong positive correlations were observed among VAR11, VAR12, VAR13, and VAR14 (with VAR13 and VAR14 reaching r = 0.777), as well as among VAR15, VAR16, and VAR17. These correlations may indicate redundancy among predictors, potentially impacting the model’s stability and interpretability.

Table 5 outlines the analysis of variance, confirming the model’s statistical significance (*p* < 0.000). The regression sums of squares (22,061,157 indicates the variance explained by the model, while the residual sum of squares (103,992.804) reflects unexplained variance.

**Table 5:** The analysis of variance.

Model	Sum of squares	df	Mean square	F	Sig.
**Regression**	22,061.15	17	1297.71	2.69	0.00
**Residual**	103,991.80	216	481.44		
**Total**	126,052.96	233			

Table 6 lists the unstandardized and standardized coefficients for each predictor. Unstandardized coefficients (B) represent the expected change in the dependent variable due to a one-unit increase in the predictor variable, provided all other variables remain constant. These values are given in the original measurement units and provide useful information about real-world relationships within the data. Standardized coefficients, also known as Beta weights, quantify the effect of an independent variable on the dependent variable in standard deviations. A one-standard-deviation rise in the predictor results in a Beta-sized change in the outcome variable. These coefficients enable direct comparison of the relative strength of predictors within a single model (Field, 2018). Two predictors emerged as statistically significant at the 0.05 level:

- VAR1 (Drought Severity): B = 3.721, SE = 1.624, β = 0.245, 95% CI [0.524–6.918], *p* = 0.023
- VAR4 (Urban Expansion): B = 3.365, SE = 1.655, β = 0.217, 95% CI [0.071–6.60], *p* = 0.045

**Table 6:** The unstandardized and standardized coefficients.

Model	B	Std. error	Beta	t	Sig.	95.0% Confidence interval for B	
						Lower bound	Upper bound
**E**	9.546	11.554		0.826	0.410	−13.227	32.320
**VAR1**	3.721	1.622	0.183	2.294	0.023	0.524	6.918
**VAR2**	0.632	1.801	0.032	0.351	0.726	−2.918	4.182
**VAR3**	−0.254	1.814	−0.012	−0.140	0.889	−3.829	3.322
**VAR4**	3.365	1.671	0.181	2.014	0.045	0.071	6.660
**VAR5**	1.703	1.815	0.087	0.938	0.349	−1.876	5.281
**VAR6**	0.836	1.389	0.049	0.602	0.548	−1.902	3.575
**VAR7**	0.809	1.764	0.042	0.459	0.647	−2.667	4.285
**VAR8**	−2.800	1.681	−0.151	−1.665	0.097	−6.114	0.514
**VAR9**	2.324	2.462	0.067	0.944	0.346	−2.529	7.178
**VAR10**	−0.723	1.748	−0.030	−0.414	0.680	−4.168	2.722
**VAR11**	−0.094	1.955	−0.005	−0.048	0.962	−3.947	3.759
**VAR12**	−0.199	1.675	−0.011	−0.119	0.905	−3.501	3.102
**VAR13**	0.877	2.063	0.047	0.425	0.671	−3.189	4.944
**VAR14**	−2.222	2.105	−0.115	−1.056	0.292	−6.371	1.926
**VAR15**	0.685	1.471	0.038	0.466	0.642	−2.214	3.583
**VAR16**	0.805	1.770	0.041	0.455	0.650	−2.683	4.294
**VAR17**	2.203	1.812	0.112	1.216	0.225	−1.369	5.776

Other variables, such as VAR8 (Soil Erosion) and VAR14 (Air Pollution), showed negative coefficients but were not statistically significant at the conventional level (*p* = 0.097 and *p* = 0.292, respectively), though their effect sizes suggest potential relevance.

**Figure 4:**
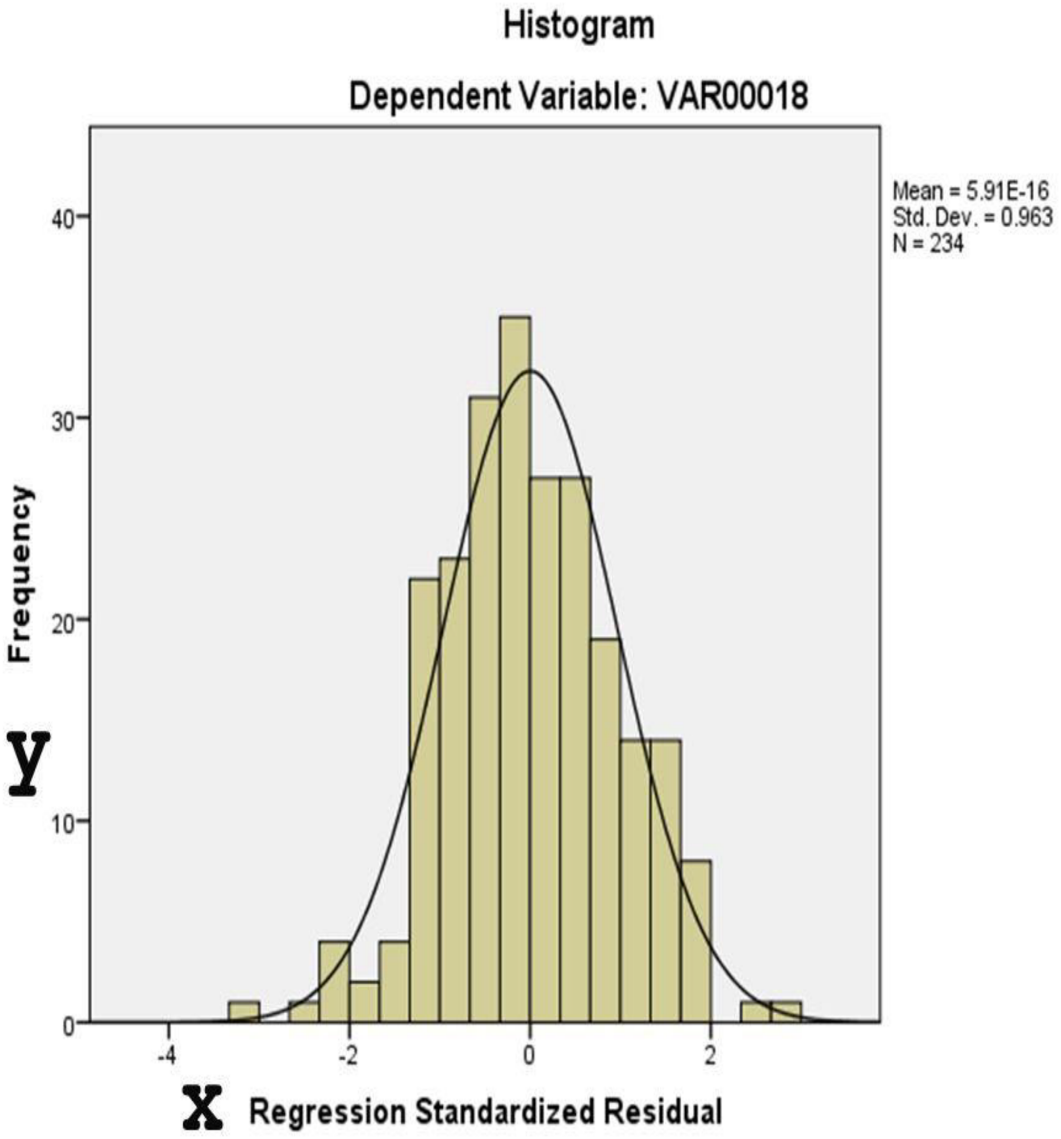
This histogram assesses the normality of residuals from the regression analysis. The distribution approximates a normal curve, supporting the assumption of normally distributed errors.

**Figure 5:**
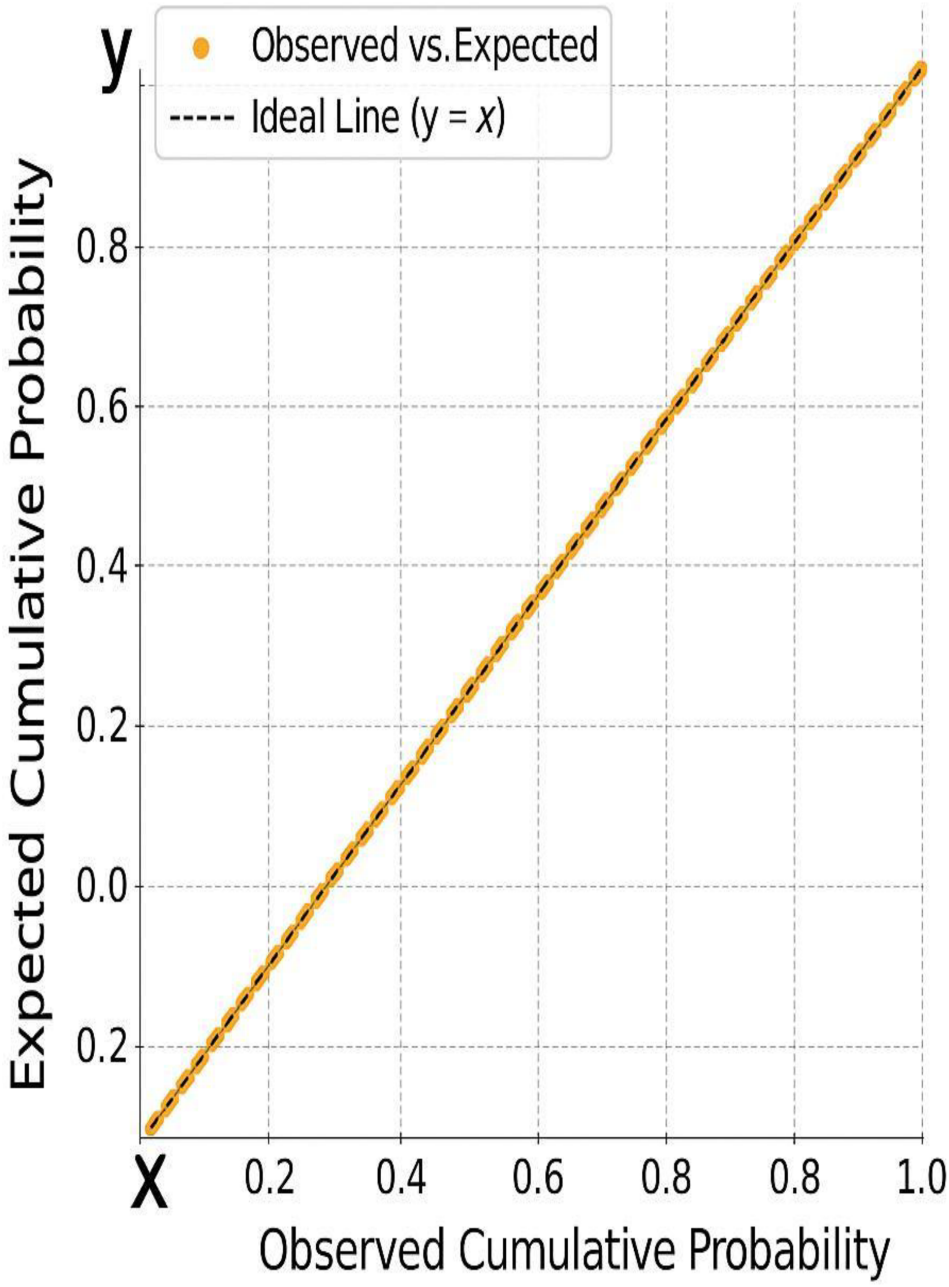
The alignment of residuals along the diagonal, further confirming the assumption of normality required for valid regression modeling.

Figures 4 and 5 display diagnostic plots to assess model assumptions:

- Figure 4: A histogram of standardized residuals confirms normality (Mean = 0, Std. Dev. = 0.963).
- Figure 5: The normal P-P plot shows points closely aligned with the diagonal line, supporting the assumption of normally distributed residuals.

**Figure 6:**
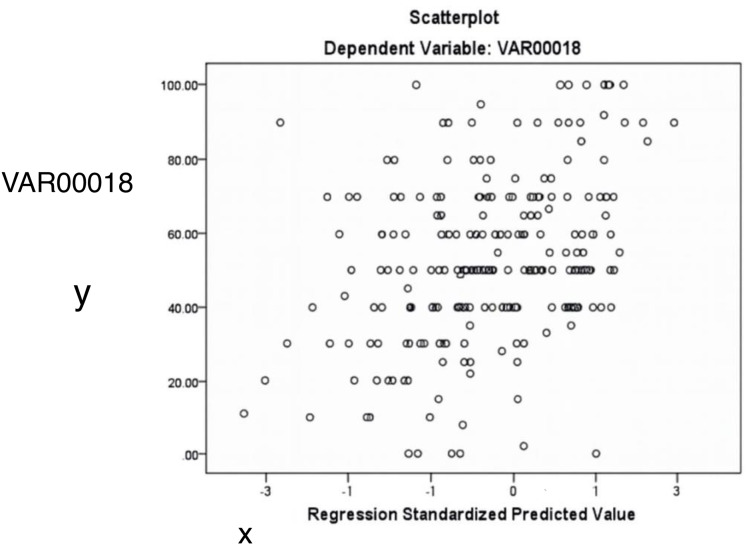
The relationship between predicted and actual values of the dependent variable (acacia decline). The random distribution of points supports the assumption of homoscedasticity (constant variance).

Figure 6 illustrates the relationship between standardized predicted and actual values of the dependent variable (VAR18). The scattered distribution indicates no apparent pattern, supporting the assumption of homoscedasticity.

Table 7 provides results for two normality tests applied to the unstandardized residuals of a dataset with 234 observations. Residual diagnostics supported key model assumptions. Both the Kolmogorov-Smirnov (*p* = 0.200) and Shapiro-Wilk tests (*p* = 0.378) indicated no significant deviation from normality. Histogram and P–P plots confirmed that the distribution of residuals was approximately normal. Additionally, the residuals were homoscedastic, as shown by the evenly scattered standardized residuals around zero, without systematic patterns.

**Table 7:** Normal tests.

	Kolmogorov-smirnov			Shapiro-wilk		
	Statistic	df	Sig.	Statistic	df	Sig.
**Unstandardized residual**	0.047	234	0.200*	0.993	234	0.378

**Note:**

Kolmogorov-Smirnov and Shapiro-Wilk tests were applied to assess residual normality. Both *p*-values > 0.05 indicate residuals follow a normal distribution.

### The results of predictive modeling

This study applies the CHAID method to develop a classification tree model. The analysis examines the relationship between a dependent variable (VAR18) and a set of independent variables (VAR1–VAR17). The CHAID algorithm partitions the dataset into statistically significant subgroups based on chi-square tests, allowing for the identification of patterns in the data.

The classification tree was constructed using the following parameters:

Parameter: Specification
Dependent variable: VAR18
Independent variables: VAR1–VAR17
Validation method: None (entire dataset used)
Maximum tree depth: 3
Minimum cases per parent node: 100
Minimum cases per child node: 50

CHAID algorithm parameters

- Splitting criterion: Chi-square test
- Significance level for splitting: 0.05 (adjusted using Bonferroni correction)
- Merging criterion: 0.05 significance level
- Handling of missing data: Nominal missing values were treated as missing cases

The CHAID decision tree consists of five nodes, including three terminal nodes, with two primary independent variables (VAR13 and VAR1) identified as the most significant predictors.

**Figure 7:**
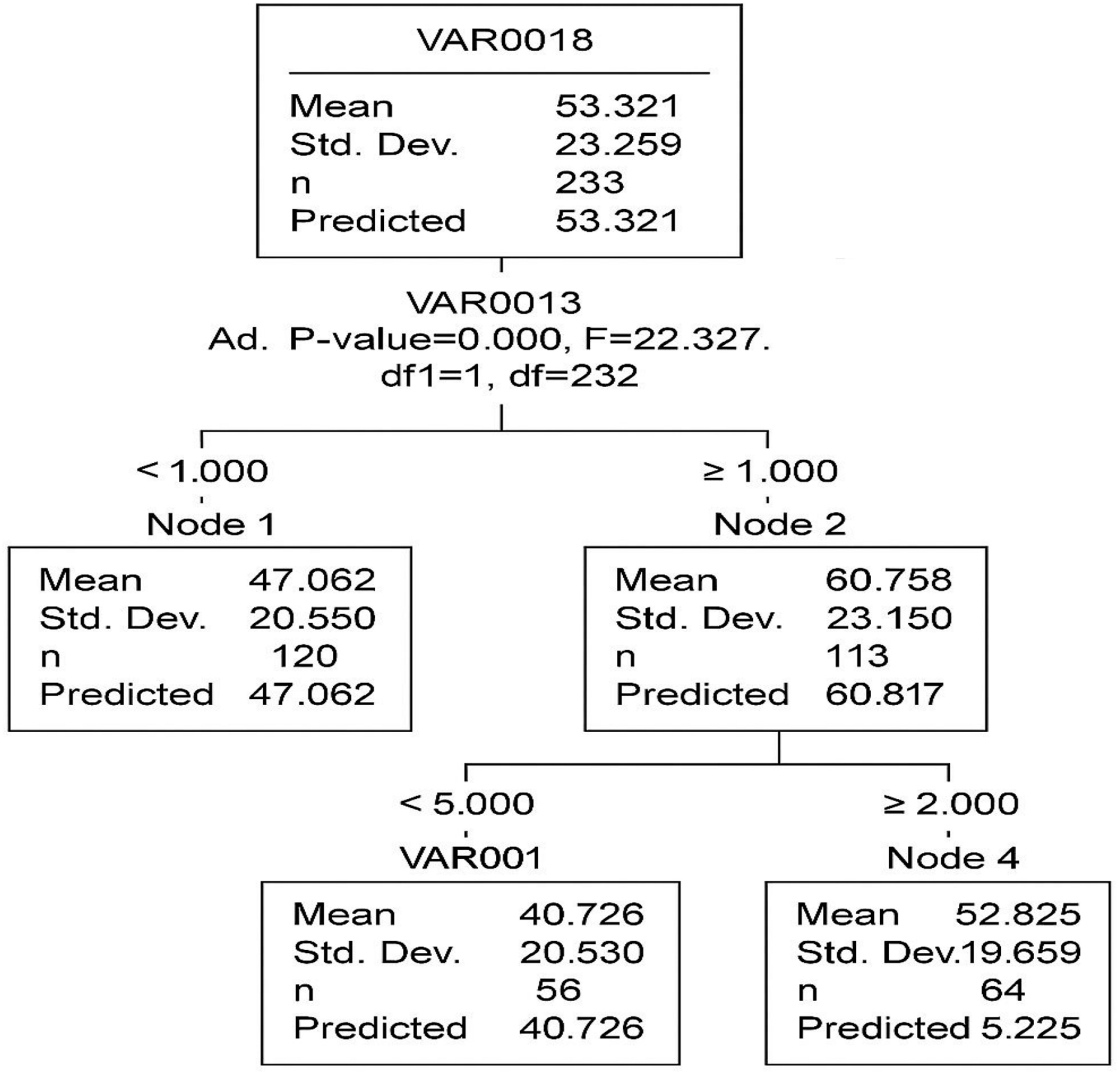
The segmentation of data based on two key predictors industrial activities (VAR13) and drought severity (VAR1). Each node represents a subgroup with a distinct mean level of acacia decline, helping to identify high-risk areas.

In Fig. 7, the root node (Node 0) represents the entire dataset with an average value of 53.321. The first significant split is based on VAR13 (*p* < 0.001, F = 22.327), creating two subgroups:

- Node 1 (mean = 47.062)
- Node 2 (mean = 60.877)

A second split occurs in Node 2, based on VAR00001 (*p* = 0.002, F = 15.784), forming two additional terminal nodes:

- Node 3 (mean = 70.120)
- Node 4 (mean = 52.625)

The model’s overall risk estimate is 456.846, with a standard error of 42.388, indicating the extent of prediction error. The gain summary (Table 8) provides insights into the effectiveness of each terminal node. Node 3 exhibits the highest mean outcome (70.120), suggesting that individuals in this category have the most distinct response to the independent variables.

**Table 8:** The gain summary.

Node	Sample size (n)	Percentage (%)	Mean
Node 3	50	21.4%	70.120
Node 4	56	23.9%	52.625
Node 1	128	54.7%	47.062

The CHAID classification tree effectively identifies VAR13 and VAR1 as the primary variables influencing VAR18. The model segments the data into distinct groups with varying mean values, offering valuable insights into the dataset’s structure. Future studies may improve the model’s accuracy through cross-validation techniques and the inclusion of additional predictor variables.

## Discussion

The results of this study underscore the multifaceted interactions between environmental conditions and anthropogenic activities in driving acacia tree deterioration. Statistical analyses confirmed that climatic variables, particularly temperature increases and reduced rainfall, have a significant negative impact on tree health. Additionally, soil degradation, manifested in high salinity and erosion, further exacerbates these challenges, corroborating previous research on vegetation loss in arid ecosystems (Zhang et al., 2003).

Human activities, particularly overgrazing and urban expansion, also emerged as critical factors contributing to acacia decline. Overgrazing prevents natural regeneration by damaging young saplings, while deforestation for firewood and urban development accelerates habitat loss. These findings align with existing literature emphasizing the detrimental effects of unregulated land use on ecological sustainability.

The predictive modeling using CHAID decision trees effectively identified high-risk zones for acacia deterioration, providing a valuable framework for targeted conservation strategies. The results indicate that intervention efforts should focus not only on mitigating environmental stressors but also on implementing policy-driven solutions.

The beetle species *Steraspis speciosa* has been recognized as a key biotic stressor causing acacia tree decline, particularly in dry locations experiencing extended drought. In Saudi Arabia, this xylophagous bug destroys trees by burrowing under the bark and disturbing xylem and phloem tissues, hindering water and nutrient flow, and speeding up tree mortality (Alanazi et al., 2022).

**Figure 8:**
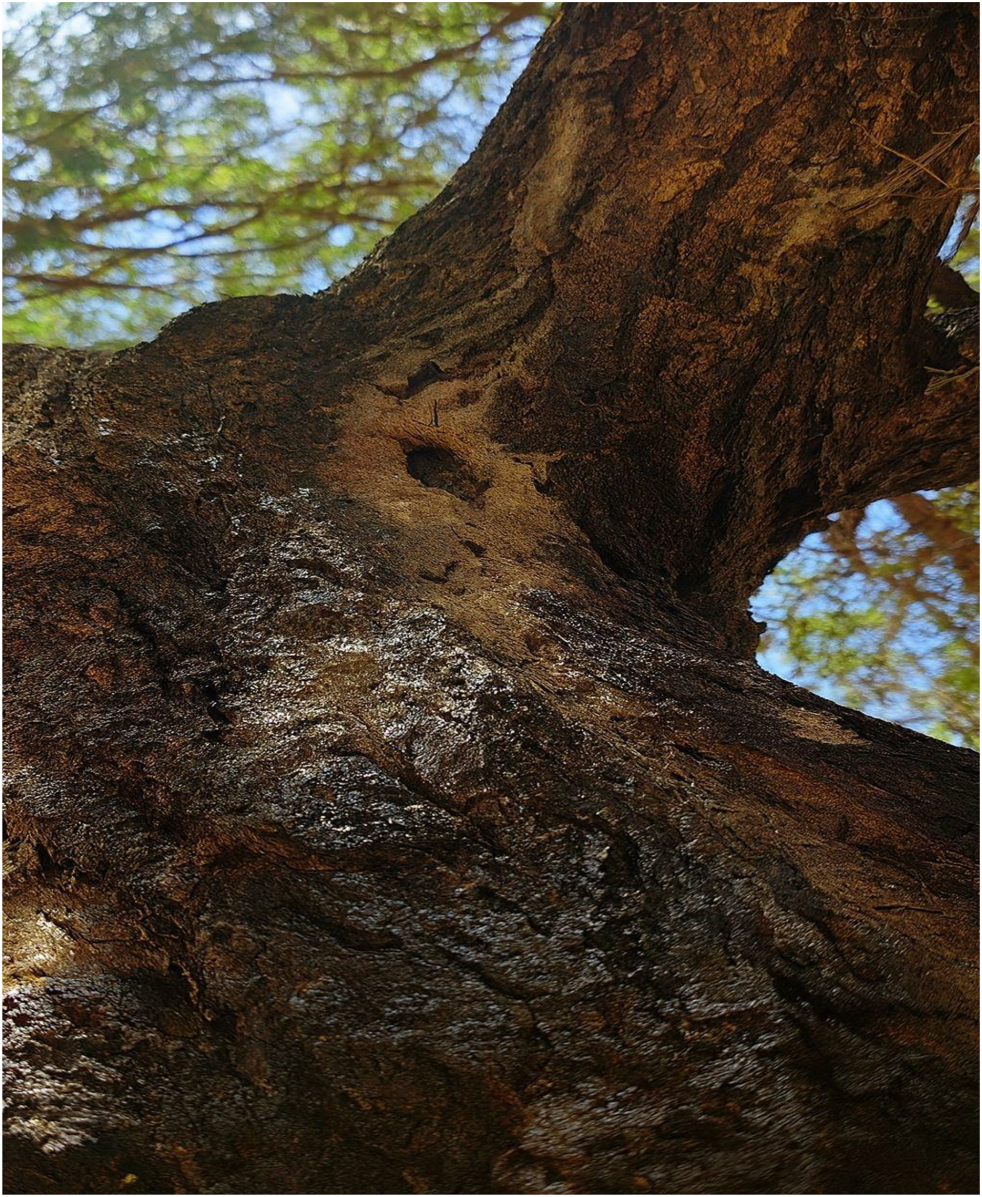
The physical damage to acacia tree bark caused by the xylophagous beetle Steraspis speciosa, including boreholes and cracking, indicative of severe pest infestation linked to environmental stress.

Figure 8 presents visual evidence of the damage caused by Steraspis speciosa to Acacia trees in the Ha’il region, characterized by darkened, cracked bark and boreholes typical of xylophagous beetle activity.

**Figure 9:**
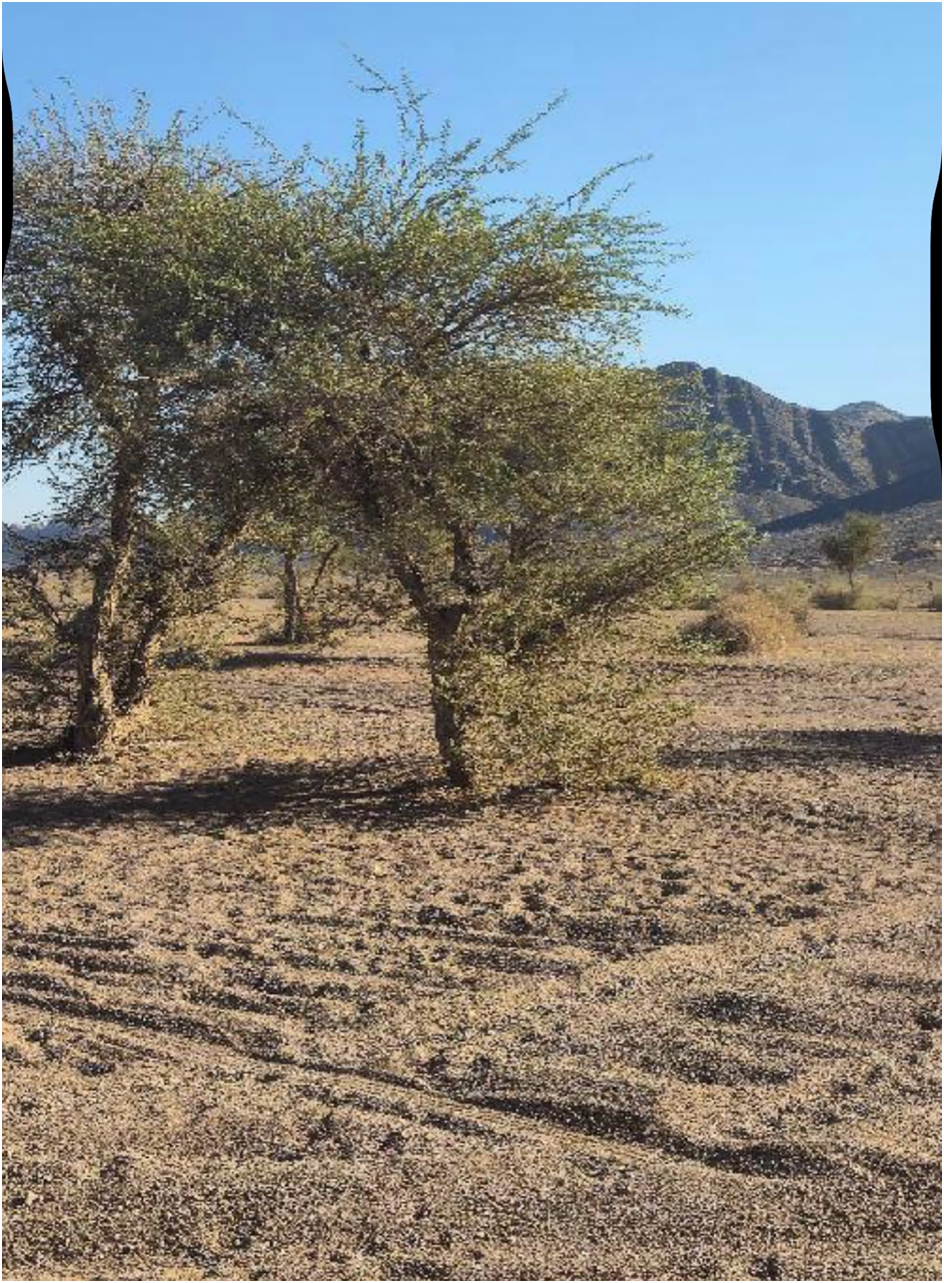
Isolated or sparsely clustered acacia trees in the arid terrain of Ha’il, reflecting sparse vegetation cover due to limited rainfall and grazing pressure.

The Ha’il region is distinguished by its dry terrain, sandy soils, and scattered vegetation. Figure 9 shows that Acacia trees are often found as lone individuals or in tiny groups, with little understory growth due to low rainfall and grazing pressure.

**Figure 10:**
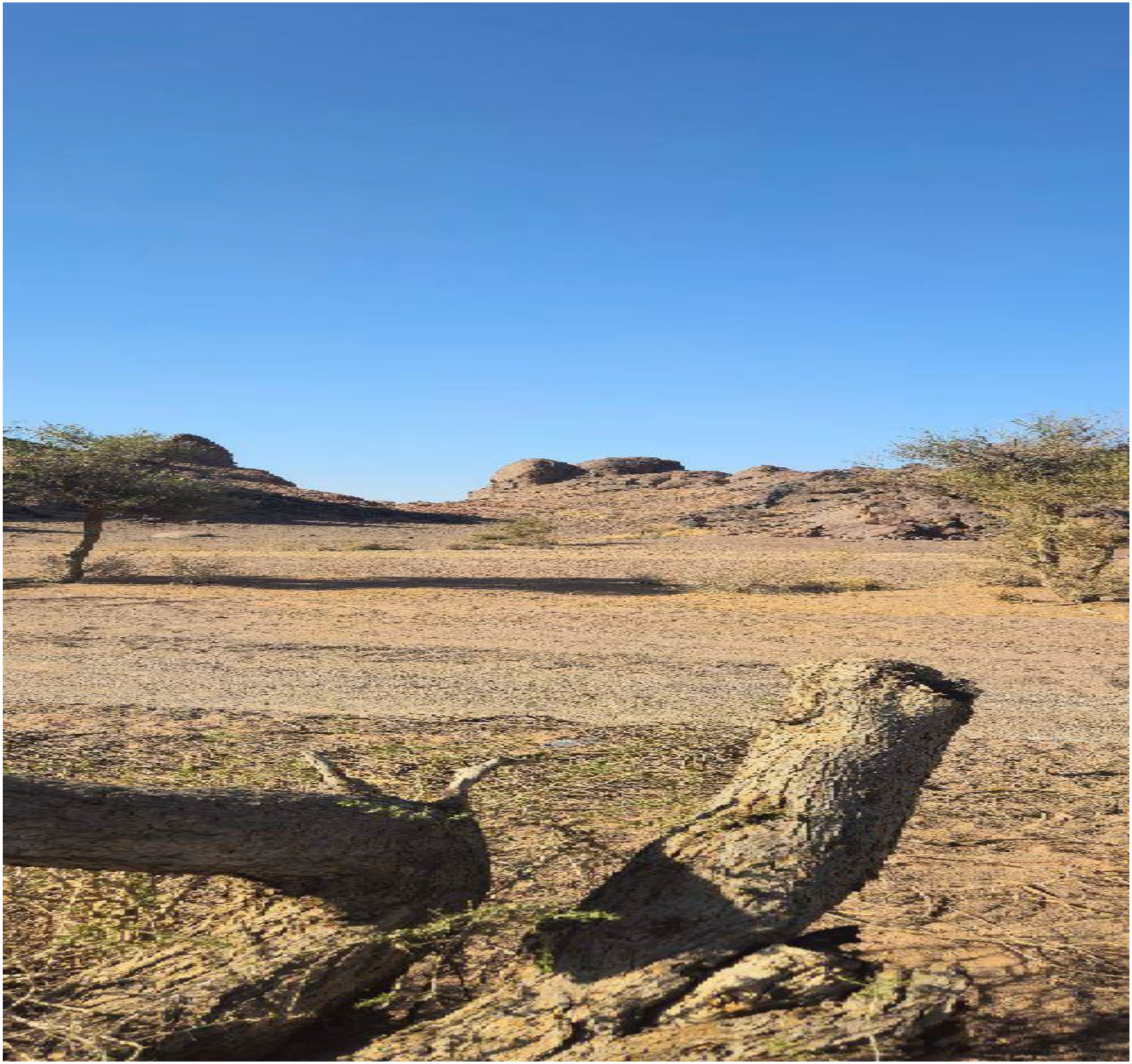
Signs of severe environmental degradation such as damaged trees, exposed rocky soils, and vegetation loss—effects attributed to overgrazing, drought, and pest activity.

As shown in Fig. 10, the research region has numerous evidence of land degradation, such as injured Acacia trees and exposed rocky soil surfaces. These patterns are consistent with the consequences of chronic drought, overgrazing, and biotic stresses like Steraspis speciosa infestation.

**Figure 11:**
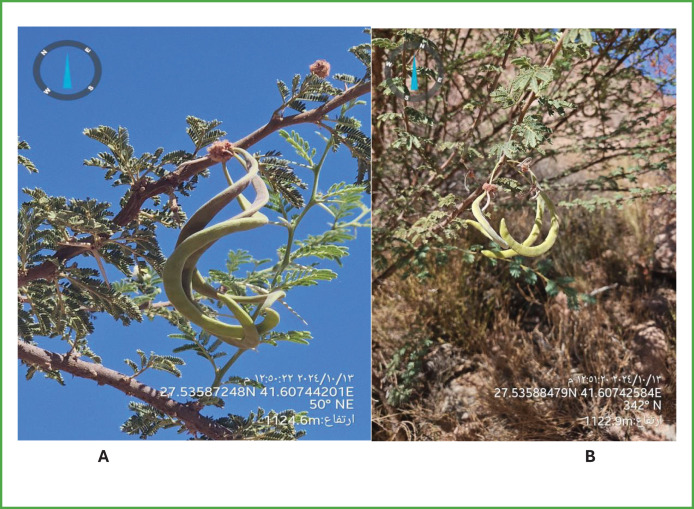
(A) A detailed view of compound leaves, spherical flowers, and seed pods. (B) A pod-bearing branch with geographic metadata included, supporting species identification and site-specific observations.

Field documentation included photographic records of Acacia morphology and reproductive structures. As shown in Fig. 11A, the plant exhibits compound leaves, spherical flower heads, and twisted seed pods characteristic of species commonly found in the Ha’il region. The image also provides geographic metadata essential for reproducibility. As part of the field survey, morphological observations of Acacia trees were recorded along with their exact coordinates and elevation data. Figure 11B presents a typical pod-bearing branch, providing further visual evidence for species identification and environmental context.

Recent advances in reforestation tactics for arid and semi-arid settings have centered on incorporating agriculture techniques that improve seedling survival and ecosystem restoration. One such option is to employ “planting units, “engineered structures that supply limited reserves of water and nutrients to nourish individual seedlings during droughts. These technologies are designed to improve water retention in the root zone, reduce evaporation, and encourage early root development, which are crucial in places like the Ha’il region, where water availability is restricted and soil conditions are poor. Incorporating these strategies throughout future Acacia rehabilitation efforts could boost planting success rates and long-term ecosystem resilience (Liiv et al., 2025).

The recognition of urban expansion and overgrazing as key drivers of Acacia tree decline in the Ha’il region aligns with findings from other arid environments, where human activities intensify ecological degradation. Urban development contributes to habitat disruption, soil compaction, and diminished water infiltration factors that collectively hinder tree establishment and regeneration. Similarly, uncontrolled grazing impairs seedling growth, disrupts natural regeneration, and accelerates soil erosion, particularly in areas without adequate land management.

Comparable outcomes have been observed in the Negev Desert, where Acacia raddiana, a keystone species, suffers from overgrazing and water scarcity, leading to declines in biodiversity and changes in soil structure (Munzbergova & Ward, 2002). In Tunisia, while *Acacia tortilis* improves soil microbial activity and organic content, these benefits are significantly reduced under intensive grazing, underscoring the importance of regulated land use (Field, 2018).

Environmental stressors such as prolonged drought, rising temperatures, and soil salinization further threaten Acacia trees by limiting water availability and nutrient uptake. These factors weaken tree vitality and increase susceptibility to pests and diseases. Similar challenges have been documented in India, where Acacia-based Silvi pastoral systems experience reduced productivity and survival under degraded land conditions, unless mitigated through sustainable land management (Yadav et al., 2014).

What distinguishes the Ha’il region is the diffuse pattern of urban expansion, likely driven by scattered rural settlements and advancing desertification, rather than centralized urban growth. This spatial pattern presents a unique ecological context compared to other arid regions.

Despite the study’s valuable insights, certain limitations should be acknowledged. The reliance on self-reported data may introduce response bias, and the absence of direct field measurements limits the ability to validate perceptions quantitatively. Future research should integrate remote sensing technology with on-the-ground ecological assessments to improve data accuracy and predictive reliability.

## Conclusion and recommendations

### Conclusion

This research demonstrates that the deterioration of Acacia trees in the Ha’il region is driven by a combination of environmental stressors and human-related activities. Key ecological pressures include prolonged drought, increasing temperatures, and soil degradation, while human influences such as unregulated urban development and excessive grazing intensify the decline. Regression results showed that drought severity and urban expansion significantly impact tree health, explaining 17.5% of the variation. Furthermore, the CHAID analysis pinpointed urban growth and overgrazing as the most influential factors, highlighting specific areas that are most at risk.

A notable strength of this study lies in its innovative methodology, merging community-based surveys, satellite and meteorological data, and advanced statistical modeling (*e.g*., regression and CHAID decision trees). This integrated approach offers a richer and more accurate picture of the challenges facing Acacia populations.

Given the scale and severity of the issue, immediate and well-informed conservation actions are essential. Recommendations include enforcing stricter land use regulations, enhancing irrigation practices, expanding reforestation and protected zones, and raising public awareness about the ecological importance of Acacia trees. The study provides a scientifically grounded roadmap for stakeholders to protect and sustainably manage these vital desert ecosystems.

### Recommendations

1. Strengthening conservation policies: Implement and enforce regulations to control overgrazing and prevent illegal logging.
2. Expanding reforestation efforts: Establish protected areas and promote large-scale acacia replanting initiatives.
3. Promoting sustainable land management: Encourage environmentally responsible agricultural and urban planning practices to minimize ecological degradation.
4. Enhancing water resource management: Develop and implement improved irrigation techniques to support tree health, particularly in vulnerable areas.
5. Raising public awareness: Conduct educational campaigns to inform local communities about the ecological and economic importance of acacia trees and promote sustainable land-use practices.
6. Advancing research and monitoring: Integrate remote sensing technology with ecological field assessments to track tree health, assess conservation effectiveness, and refine predictive models.

By implementing these measures, policymakers, conservationists, and local stakeholders can work collaboratively to mitigate the decline of acacia trees and ensure their long-term sustainability in arid and semi-arid ecosystems.

## Supplemental Information

10.7717/peerj.20067/supp-1 Supplemental Information 1 Raw Data.

## Additional Information and Declarations

### Data Availability

The following information was supplied regarding data availability:

The raw data is available in the Supplemental File.
